# Bioactive Compounds from Cruciferous Vegetables as a Therapeutic Option for the Prevention and Treatment of Cardiovascular Diseases

**DOI:** 10.3390/nu18050810

**Published:** 2026-03-01

**Authors:** Beata Olas

**Affiliations:** Department of General Biochemistry, Faculty of Biology and Environmental Protection, University of Lodz, Pomorska 141/143, 90-236 Lodz, Poland; beata.olas@biol.uni.lodz.pl; Tel./Fax: +48-42-6354485

**Keywords:** cardioprotective activity, cruciferous vegetables, pro-healthy potential

## Abstract

Vegetables, including cruciferous vegetables, contain a variety of active compounds with cardioprotective potential, for example fiber, minerals, and phytochemicals such as phenolic compounds, terpenes, carotenoids, and others. Cruciferous vegetables are also particularly rich in sulfur-containing compounds such as glucosinolates, which have cardioprotective effects. However, there is little information about the molecular mechanisms of their action. This paper reviews the current state of knowledge regarding the cardioprotective capacity of cruciferous vegetables; it also examines their chemical composition and the mechanisms behind this biological property. In this narrative review, the author also summarizes data on changes in the content of various bioactive compounds (especially phenolic compounds, carotenoids, and glucosinolates) and their biological properties, including cardioprotective efficacy during vegetable processing (for example, lactic acid fermentation, cooking and other).

## 1. Introduction

Various studies indicate medicinal benefits of consuming fruits and vegetables. There is also evidence that high consumption of fruits and vegetables plays a central role in prevention of cardiovascular diseases (CVDs) [1,2,3,4,5,6,7,8,9,10]. Some studies note an inverse relationship between consumption of cruciferous vegetables (*Cruciferae* or *Brassicaceae*) (named for their four equal-sized petals in the shape of a “crucifer” cross) and risk of CVDs [2,8,11,12,13,14,15,16,17,18,19]. For example, Murashima et al. [19] found that intake of broccoli sprouts (100 g/day, for one week) not only improves cholesterol metabolism but also decreases oxidative stress in twelve healthy subjects.

Vegetables, including cruciferous vegetables, contain a variety of active compounds with cardioprotective potential, for example fiber, minerals, and phytochemicals such as phenolic compounds, terpenes, carotenoids, and others. Cruciferous vegetables are also particularly rich in sulfur-containing compounds such as glucosinolates, which have cardioprotective effects [17,20,21,22,23,24]. In addition, the cardioprotective potential may also be associated with the consumption of nitrate-rich vegetables, including cruciferous vegetables [9].

Cruciferous vegetables originated from the Irano-Turanian region about 20 million years ago. They include various economically important species, mainly vegetable species, edible oil plants, feed plants, and spice plants (for example, *Brassica oleracea* var. *italica* (broccoli), *Brassica oleracea* var. *botrytis* (cauliflower), *Brassica oleracea* var. *capitata* (cabbage), *Brassica oleracea* var. *sabellica* (kale), *Brassica oleracea* var. *Gemmifera* (Brussels sprouts), *Brassica oleracea* var. *gongylodes* L. (cabbage), *Brassica rapa* (turnip), *Brassica napus* (rape)*, Sinapsis* L. (mustard), *Raphanus sativus* (radish), *Lepidium sativum* L. (cress), *Nasturtium officinale* (watercress), *Armoracia rusticana* (horseradish), and *Eruca vesicaria* (L.) Cav. (arugula)). Moreover, cruciferous vegetables and their products are very nutritive. These vegetables may be consumed in a diet in various forms: in the form of a fresh salad and steamed. According to the Food and Agriculture Organization (FAO), cabbage (known as a major cruciferous vegetable) had an annual global production of 72,604 kilotons in 2022. Common names of cruciferous vegetables and parts used for human consumption as vegetables are described in Table 1. In addition, cruciferous vegetables also fall into the “dark-green vegetables” category which includes kale, broccoli, mustard greens, and other vegetables (for example, cabbage) category [5,25].

Although various papers, especially systematic reviews and meta-analysis, indicate that cruciferous vegetables have health benefits [1,2,3,4,5,6,7,8,9,25], these present little information about the key ingredients with cardioprotective activity. These review articles generally do not include information about their cardioprotective mechanisms [2,8,12,13,14,15,17,18,26]. For example, Chen et al. [27] observe that increased consumption of cruciferous vegetables promotes cardiovascular health and reduces related mortality. These observations were based on results from the Singapore Chinese Health Study and meta-analysis. Madsen et al. [8], using meta-analysis, also note positive associations between high consumption of cruciferous vegetables and reduced risk of hypertension. The same results were noted by Connolly et al. [23].

Preparations from cruciferous vegetables have high levels of different active compounds with various biological activities, but strong scientific evidence of their cardioprotective efficacy is still lacking. Therefore, for the first time, in this narrative review, the present work analyses the up-to-date literature concerning the effect of active compounds (phenolic compounds, glucosinolates, and others) of cruciferous vegetables on selected factors of CVDs. In this narrative review, the author also summarizes data on changes in the content of various active compounds (especially phenolic compounds, carotenoids, and glucosinolates) and their biological properties, including cardioprotective efficacy during vegetable processing (for example, lactic acid fermentation, cooking and other).

## 2. Methodology for the Literature Search

A literature search of PubMed, Science Direct, Scopus, Springer, Web of Knowledge, Web of Science, and Google Scholar was performed, using various combinations of the keywords: “cruciferous”, “vegetables”, “cruciferous vegetables”, “cardioprotective activity”, and “cardiovascular disease”. No time criteria were applied to the search, but recent papers were evaluated first. All papers were imported to Mendeley Reference manager. The identified articles (198 articles) were first screened by reading the abstracts. After obtaining the full texts of the included studies, the reference sections were also manually examined to identify any additional new articles. The last search was run on 30 December 2025. Only the studies that measured the cardioprotective properties of bioactive compounds (phenolic compounds, glucosinolates, and others) from cruciferous vegetables were included. Moreover, both in vitro and in vivo studies were taken into consideration. Only studies in English and Polish were included.

Data extracted from each article were: the type of studied material (species, cultivar, or other relevant data), methods used in the study/study design, number of replicates, cardioprotective activity, and statistical significance.

## 3. Cardioprotective Potential of Bioactive Compounds from Cruciferous Vegetables

The active compounds found in cruciferous vegetables are not only responsible for smell, taste, and giving color, but they have also been demonstrated to have various positive effects on human health [21]. Cruciferous vegetables are also foods rich in nutritive composition. In addition, they are a good source of fiber, which has cardioprotective activity. For example, among these vegetables, the content of kale is richer in fiber (4.1 g/100 g) than other cruciferous vegetables (arugula (1.6 g/100 g), cauliflower (2 g/100 g), broccoli (2.4 g/100 g), and cabbage (2.5 g/100 g)). Cruciferous vegetables are also good dietary sources of potassium, magnesium, phosphorus, iron, calcium, vitamins, especially vitamin C and K, carotenes, and folic acid. Other bioactive compounds found in these vegetables are phenolic compounds and fatty acids (stearic, palmitic, oleic, linoleic, linolenic, and others). Phytosterols are the other bioactive compounds with cardioprotective properties presented in cruciferous vegetables, but the amount of these ingredients varies according to the type of cruciferous vegetable [2,24,28,29,30].

### 3.1. Phenolic Compounds

A major group of bioactive components present in cruciferous vegetables are phenolic compounds, which refer to a large group of phytochemicals that comprise an aromatic ring bearing on or more hydroxyl substituents. The most diverse group of phenolic compounds in cruciferous vegetables are phenolic acids (for example, ferulic, sinapic, and caffeic acids), flavonoids (for example, quercetin, and cyanidin), flavonols, flavones, coumarins, tannins, and, in red vegetables (red cabbage, purple cauliflower, purple pak-choi, red curly kale, and red radishes), anthocyanins that affect biological properties, especially the antioxidant potential of these vegetables.

In vitro and in vivo models have demonstrated that phenolic compounds presented in cruciferous vegetables exert cardioprotective action via different pathways, including not only controlling oxidative stress, but also inflammation [20,21,22,23,24,25]. Phenolic compounds are also considered to contribute to other health benefits associated with the consumption of cruciferous vegetables such as anti-platelet activity. Especially, dietary intake of anthocyanins is linked to reduced risks of CVDs [31,32,33,34,35,36,37]. However, the profile of phenolic compounds can vary in the organs of the same plant; for example, cruciferous sprouts contain from 2 to 10 times more phenolic compounds when compared with their roots [31,36].

Zhang et al. [37] noted that, in fresh cruciferous vegetables, the phenolic compounds present are very often highly acylated and glycosylated, forming complex molecules. Moreover, Li et al. [34] found that the major flavonoids presented in these vegetables are glycosylated isorhamnetin, kaempferol, and quercetin.

According to various studies [31,32,33], broccoli sprouts possess more antioxidants, including phenolic compounds, than other cruciferous. In other experiments, phenolic compound content of selected commercially available cruciferous vegetables, including Chinese cabbage, red cabbage, green cabbage, mustard cabbage, and Chinese white cabbage, and their antioxidant capacity, were evaluated. This study indicates that red cabbage possesses the highest phenolic compounds content and antioxidant capacity among all tested vegetables [38,39,40].

Recently, Yeo et al. [41] have noted that novel purple Chinese cabbages (85,772 and 65,065; derived from interspecific hybridization) are favorable alternatives to the typical green Chinese cabbage, given not only the higher content of phenolic compounds, but also carotenoids.

#### 3.1.1. In Vitro Models

Li et al. [34] evaluated the total phenolic content and antioxidant activity of twelve cruciferous vegetables (watercress, pakchoi, daikon radish, choysum, red cherry radish, Chinese cabbage, rocket salad, kalian, broccoli, Brussels sprout, cauliflower, and cabbage) in vitro. A total of seventy-four phenolic compounds were identified, including fifty-eight flavonoids and derivatives, and 16 hydroxycinnamic acids. In addition, Spearman’s correlation demonstrated significant positive correlation between total phenolic compounds and antioxidant properties. Brussels sprouts (26.7 ± 10.5 µmol TE/g FW), watercress (32.9 ± 1.7 µmol TE/g FW), and rocked salad (32.1 ± 7.5 µmol TE/g FW) possess the highest antioxidant activity measured by oxygen radical absorbance capacity (ORAC), followed by kailan (23.7 ± 4.9 µmol TE/g FW), while Chinese cabbage (3.4 ± 0.2 µmol TE/g FW), cabbage (7.0 ± 1.5 µmol TE/g FW), and daikon radish (5.3 ± 2.5 µmol TE/g FW) possess the lowest. These results are in agreement with other studies by Kaur and Kapoor [40].

Recently, Zheng et al. [42] isolated and characterized bioactive constituents from the methanolic extract of *E. sativa* leaves. They also evaluated cardioprotective potential and antiplatelet properties of these bioactive compounds in two pathologically relevant models: (1) production of reactive oxygen species (ROS) and apoptotic cell death in cardiomyocytes under hypoxia/reoxygenation conditions, and (2) ROS-mediated blood platelet activation, including platelet aggregation. Among them, an isolated compound from *E. sativa*, compound 2 (kaempferol glycoside; 0.5–2 mM), had cardioprotective action and anti-aggregatory properties (using washed blood platelets (5 × 10^8^/mL)). For example, compound 2 inhibited a generation of ROS in both cardiomyocytes and blood platelets, and attenuated hypoxia-/reoxygenation-induced apoptosis. Authors suggest that compound 2 reduces ROS generation during blood platelet activation–aggregation through NADPH oxidase (NOX) inhibition or scavenging of hydrogen and superoxide peroxide.

Bhatt et al. [43] observed that a cold water extract (in which flavonoids and polyphenolics were identified) of cauliflower has antioxidant properties of the 2,2-diphenyl-1-picrylhydrazyl (DPPH) scavenger. Other in vitro studies identified nine phenolic acids (caffeic acid and ferulic acid being the most abundant) in kale, and these compounds increased DPPH scavengers [43]. In addition, various radish extracts (50 and 100 µg/mL) with butanol, chloroform, ethyl acetate, hexane, and water-soluble fractions had anti-inflammatory and anti-platelet potential (in vitro) [44].

Saluk et al. [45] investigated the potential protective properties of red cabbage anthocyanins (0.15–1.5 µg/mL, which are recognized as the most important polyphenolic components present in fresh and pickled red cabbage) against oxidative damage induced by lipopolysaccharides (LPSs) in blood platelets (in vitro model). They observed that the tested extract effectively decreases oxidative stress induced by LPSs. Moreover, the in silico analysis showed that both cyanin and LPS were located at the same region of the human TLR4-MD-2 complex. Other results also demonstrated the antioxidant potential of red cabbage anthocyanins in in vitro models [46,47].

#### 3.1.2. In Vivo Models

Results of Sharma et al. [48] indicate that a phenolic-rich extract of cabbage (*B. oleracea* var. *gongylodes*, 800 mg/kg body weight) has not only antioxidant activity, but also antihyperlipidemic properties in Wistar rats (n = 5, in vivo model). For example, this extract normalized the lipid profile and improved antioxidant status—enzymatic activities of superoxide dismutase and catalase were significantly increased. The phenolic-rich extract of cabbage was given orally daily for 8 days. The major phenolic compounds present in the tested extract were chlorogenic acid, rutin, and sinapic acid.

Other in vivo results indicate that the methanolic extract from *E. sativa* Mill. has antihypertensive action (in rats). This antihypertensive action is mainly due to its vasodilatory and partly cardiac effects. Phytochemical analysis demonstrated that the tested extract was rich, especially in flavonoids [49]. In addition, a methanolic extract of leaves of *E. sativa* had anti-inflammatory [50], anti-platelet, and antithrombotic activities [51]. In the experiment of Fuentes et al. [51], an *E. sativa* aqueous extract (0.1 to 1 mg/mL) was added to human blood platelets, and different parameters of platelet activation were measured, including P-selectin expression by flow cytometry, blood platelet aggregation induced by various agonists, and thromboxane B_2_ release. In addition, antithrombotic activity of the tested extract (200 mg/kg) and bleeding time in murine models were evaluated. The *E. sativa* extract (0.1 to 1 mg/mL) had not only anti-platelet properties in vitro, but also, in murine models, the *E. sativa* extract showed significant antithrombotic activity and a slight effect on bleeding time.

### 3.2. Glucosinolates

Besides characteristic phenolic compounds, another important group of bioactive compounds in cruciferous vegetables are organosulfur compounds—glucosinolates (sulfur-rich, anionic, and water-soluble secondary metabolites) and their isothiocyanates. Glucosinolates include glucoraphanin (sulforaphane), glucobrassicin, glucoiberin, glucoraphasatin, and sinigrin. They are responsible for the bitter taste and pungent odor found in cruciferous vegetables [23,52,53,54]. In their basic chemical structure, there is an amino-acid-containing side chain that is in a sulphated isothiocyanate group linked to D-thioglucose. In cruciferous vegetables, about 95 different glucosinolates have been described. Glucosinolates are divided into three groups according to the various amino acid precursors in their side chains: indole (tryptophan), aromatic (tyrosine and phenylalanine), and aliphatic (valine, leucine, isoleucine, and methionine) [23,52,53,54].

During chopping, cutting or chewing food that contains glucosinolates, the hydrolysis of glucosinolates via enzymes (β-thioglucosidase and myrosinose) occurs, due to cellular breakdown. This results in the formation of various products, for example, isothiocyanates, thiocyanates, and nitriles. Metabolism of glucosinolates can also occur by gut microbiota. It is noted that bacterial microflora of the human colon demonstrates myrosinase activity [23,52,53,54].

About 200 various glucosinolates are known in cruciferous vegetables, but not all are commonly consumed by humans [23,52]. All the glucosinolates combined make up about 0.1–0.6% of the dry weight of these vegetables, and S-methyl cysteine sulfoxide, a non-proteinogenic sulfur-containing cysteine derivative, contributes more, at about 1–4% of the dry weight [24]. The highest known dietary sources of S-methyl cysteine sulfoxide include Brussels sprouts (≤420 mg/100 g fresh weight), and cauliflower (≤285 mg/100 g fresh weight). Moreover, these compounds are also identified in other cruciferous vegetables such as kale, cabbage, and broccoli. The level of these compounds in vegetables is influenced by environmental factors, growing, cultivar, and plant genetics. It is important that the S-methyl cysteine sulfoxides are urinary biomarkers, to indicate dietary cruciferous vegetable intake in humans [24,55,56].

Typically, cruciferous vegetables are not consumed immediately after harvesting. On the other hand, fresh these vegetables have the highest concentration of glucosinolates, but it decreases with the length of storage. For example, the glucosinolate concentration of vegetables stored at room temperature (for 5 days) decreased by about 80% compared to fresh vegetables [52,57,58]. In addition, Casajus et al. [59] also observed that storage in darkness decreased the content of aliphatic glucosinolates.

Research has shown that various glucosinolates, including glucoraphanin, decrease the risk of CVDs [60]. Glucoraphanin is an important non-toxic compound found in cruciferous vegetables, with about 80% bioavailability. For example, at 8 h, cumulative excretion was more than 50% of the ingested dose. The highest concentration of glucoraphanin was observed in the mature head stage, with a subsequent decrease as flowering begins [37].

#### 3.2.1. In Vitro Models

In an in vitro model, glucoraphanin prevents oxidized low-density lipoprotein (LDL)-induced ROS production, NF-κB nuclear translocation, vascular cell adhesion protein 1 (VCAM), ICAM, and E-selectin expression. Moreover, it has been noted that this compound induces the expression of various antioxidant enzymes [61,62,63,64,65]. In addition, glucoraphanin (15–75 µM) also had anti-platelet properties in a human blood platelet (in vitro model) [66,67].

#### 3.2.2. In Vivo Models

In various preclinical models, glucoraphanin inhibits the NF-κB DNA-binding activity and downregulates TNF-α-mediated induction of intracellular adhesion of molecule 1 (ICAM-1) in endothelial cells, suppressing inflammation in atherosclerotic lesions [61,62,63,64].

Shehatou et al. [65] observed that supplementation with glucoraphanin (0.25 mg/kg/day, for 4 weeks) protects against elevation of total cholesterol and LDL cholesterol in a rabbit model of hypercholesterolemia. Authors also indicate that glucoraphanin (0.25 mg/kg/day) ameliorates a high-cholesterol diet by 1%—it induced atherosclerosis lesion progressions and vascular dysfunction in hypercholesterolemic rabbits, probably by its antioxidant effects, lipid-lowering, and suppression of NF-κB-mediated inflammation.

Other in vivo results demonstrate that glucoraphanin (0.125 and 0.250 mg/kg) has an anti-platelet effect, including in thrombus formation. This action was observed in a thrombotic model (in vivo, (mice)) [66,67].

In an in-animal model (obese mice), glucoraphanin (5 mg/kg/day, for 14 days) had anti-obesity properties by reversing leptin resistance [68]. Ruhee and Suzuki [22] found that the prevention of obesity by glucoraphanin is also associated with decreased expression of peroxisome proliferator-activated receptor gamma (PPARγ), CCAAT-enhancer binding protein α (C/EBPα) levels, and increased levels of adiponectin, mediated by AMP-activated protein kinase (AMPK) activation. Glucoraphanin also decreased body weight and lipid profile in rodents [69]. In addition, Shawky et al. [70] suggest that glucoraphanin (0.5 mg/kg/day, for 3 weeks) may provide a rational prophylactic approach to target restenosis after angioplasty in diet-induced obesity. This action was observed in fed obese C57BL/6J mice (n = 25). More details about it are described in another review article [22].

Murashima et al. [19] note that the consumption of fresh broccoli sprouts (100 g/day, for one week) improves cholesterol metabolism and decreases oxidative stress (measuring by various biomarkers such as 8-isoprostanes, hydroperoxides, and others) in healthy human subjects (n = 12). Authors suggest that biological action of this supplementation may be associated with the presence of glucoraphanin in broccoli sprouts. The edible portion of mature broccoli contains 507–684 μg glucoraphanin/g dry matter, while broccoli sprouts contain 10 times greater glucoraphanin concentration (1153 mg/100 g dry weight) [22].

In another in vivo experiment, Armah et al. [71] studied the effect of a diet rich in high-glucoraphanin broccoli (400 g per week, for 12 weeks) on the level of plasma low-density lipoprotein (LDL) cholesterol in healthy volunteers (n = 130). This broccoli contained 21.6 ± 1.6 µmol/g dry weight glucoraphanin and 4.5 ± 0.34 µmol/g dry weight glucoiberin. The authors observed that the used diet reduces the level of LDL cholesterol. The probable mechanism by which glucoraphanin decreases LDL cholesterol is through the induction of nuclear factor [erythroid-derived 2]-like 2 (Nrf2)-antioxidant response-element-mediated transcription by glucoraphanin derived from glucoraphanin. Moreover, Nrf2 expression is associated with modulating mitochondrial fatty acid oxidation and lipid and steroid synthesis. Armah et al. [71] also suppose that other components (such as fiber, S-methylocysteine, and plant stanols) found in broccoli may reduce the level of LDL cholesterol by the inhibition of its synthesis.

Results of Melaga et al. [72] demonstrate the protective action of Tuscan black cabbage sprout extract (enriched in glucosinolates) against serum lipid increase (including total cholesterol) in rats fed a high-fat diet. The used dose of extract, 15 mg/kg bw, contained an amount of glucosinolates comparable with the average daily intake in humans.

Bahadoran et al. [73] found that broccoli extract (enriched in glucosinolates) in tablets (5 or 10 g/day, for 4 weeks) decreases LDL cholesterol and the level of markers of oxidative stress (for example, malondialdehyde (MDA) and total antioxidant capacity (TAC) serum) in patients with type 2 diabetes (n = 25).

Huang et al. [64] also noted that 1.09% red cabbage microgreens (for 8 weeks) modulate weigh gain and cholesterol metabolism in mice fed a high-fat diet (n = 60). Major active components (anthocyanins, and glucosinolates) are in red cabbage microgreens, which are used for the formulation of the animal diet. Red cabbage microgreens were added into the diet in the form of dry powder.

Other in vivo experiments found that glucoraphanin improves endothelial function and blood pressure in women (n = 12) with pregnancy hypertension. These women supplemented four capsules of the activated broccoli seed extract BroccoMax^®^ (equivalent to 32 mg of glucoraphanin, per day, for 2 weeks) [74]. Glucoraphanin administration (10 µmol/kg body weight/day, for 4 months) also normalized the kidney epigenome and improved blood pressure in hypertensive rats (n = 6) [75]. On the other hand, Christiansen et al. [76] found that daily consumption of 10 g of dried broccoli sprouts (for 4 weeks) did not improve endothelial function in humans with hypertension (n = 40). The sprouts had a glucoraphanin content of 25.9 ± 8.5 µmol/g in dry weight and a total glucosinolate content of 48.5 ± 14.2 µmol/g in dry weight.

Organic isothiocyanates have also been studied extensively in the prevention and treatment of CVDs. Phenethyl isothiocynate is one of the most studied isothiocyanates. It is a major constituent of watercress and other cruciferous vegetables. For example, Gwon et al. [77] observed that phenethyl isothiocyanate (30 and 75 mg/kg/day, for 12 weeks) protects against high fat/cholesterol diet-induced obesity and atherosclerosis in C57BL/6 mice (n = 10). This compound stimulated the reverse cholesterol transport pathway, reduced lipid accumulation, and the inflammatory response, by modulating PPARɤ, liver-X-receptor α (LXR-α), ATP binding cassette subfamily A member 1 (ABCA1), scavenger receptor A1 (SR-A1), cluster of differentiation 36 (CD36), and NF-κB. In addition, phenethyl isothiocyanate had beneficial effects on atherosclerosis and obesity via histone modification.

### 3.3. Other Components

Cruciferous vegetables are a good source of carotenoids, although the color of carotenoids is masked by chlorophyll [38,78,79]. For example, kale is one of the best sources of carotenoids among vegetables, with β-carotene and lutein being the most abundant. The quantity of carotenoids in kale depends on environmental factors during growing and the maturity stage. In other studies, Kurilich et al. [80] compared carotenoid content of various cruciferous vegetables, including broccoli, cabbage, kale, Brussels sprouts and cauliflower, and noted that kale contained the highest amount of β-carotene. In addition, these authors found that kale contains higher amounts of α-tocopherol than other tested vegetables.

In fresh curly kale juice, β-carotene and lutein accounted for 35% and 40% of total carotenoids, respectively. However, fermentation resulted in a 17–31 decrease in their content [81]. Odondo et al. [82] received similar results for raw and fermented Ethiopian kale. They identified β-carotene, zeaxanthin and lutein in raw samples. After fermentation, the total content of carotenoids changed; the concentration of zeaxanthin increased, but the total concentration of carotenoids decreased by 75% (for lutein—98%).

Moreover, it is important that cruciferous vegetables (for example, red cabbage microgreens) have beneficial effects on human health via probiotics (for example, when fermented) [83,84]. Probiotics and prebiotics may serve as important dietary components, especially in the prevention of CVDs. For example, probiotics decrease cholesterol levels and may protect against CVDs by increasing bile salt synthesis and bile acid deconjugation. Moreover, probiotics also have anti-oxidative, anti-platelet and anti-inflammatory properties [85].

Recently, Wu et al. [84] have demonstrated that the consumption of red cabbage microgreens in a diet can alter gut microbiota, and attenuation of high-fat-diet-induced body weight gain and altered cholesterol metabolism may be mediated through regulation of gut microbiota. For example, authors have observed that red cabbage microgreens significantly inhibit high-fat-diet-induced elevation of the genus *AF12*, whose abundance is positively correlated with body weight gain.

A diet rich in nitrate (NO_3_^−^) from various vegetables, including cruciferous vegetables (for example, Chinese cabbage, cabbage, broccoli, cauliflower, radish, and other), may have cardioprotective effects [9]. However, no studies have examined relationships between their cardioprotective action and supplementation with NO_3_^−^-rich cruciferous vegetables. Therefore, there is a need for further various studies examining the cardioprotective capacity of nitrate-rich cruciferous vegetables like radishes (which have a high concentration of NO_3_^−^ (total nitrate content: 1000–2500 mg/kg)). More details about the role of nitrate-rich vegetables (especially beetroot products) in prophylaxis and the treatment of CVDs are described by Olas [9].

Other components of cruciferous vegetables (for example, selenium, phytosterols and fiber) may also play a protective function in CVDs. However, it is unknown whether these components are more effective for prophylaxis and treatment of CVDs than phenolic compounds and glucosinolates. This matter needs further research, especially in large clinical studies.

The cardioprotective efficacy of various preparations of cruciferous vegetables and their active compounds is summarized in Table 2. However, these studies demonstrate considerable heterogeneity, making it difficult to compare results. The conclusions are based on studies conducted with a wide range of preparations (especially extracts and their selected bioactive compounds), with different doses, experimental models, and large methodological heterogeneity. For example, glucoraphanin was supplemented between 0.125 and 5 mg/kg/day (for 2 or 4 weeks), but there is no information about its biological properties during longer supplementation. Moreover, genetic variability, lifestyle variables, and dietary background are only sometimes noted by authors.

It is important that several articles noted cardioprotective effects of supplementation with cruciferous vegetables (especially with broccoli) in healthy humans, and humans with hypertension or patients with type 2 diabetes, but there are no studies with a large sample size. In addition, there are no clinical studies for the interaction of this supplementation with various drugs used in prophylaxis and treatment of CVDs, or the safety of cruciferous vegetables during longer consumption.

## 4. Cardioprotective Mechanisms of the Main Components of Cruciferous Vegetables

**Figure 1:**
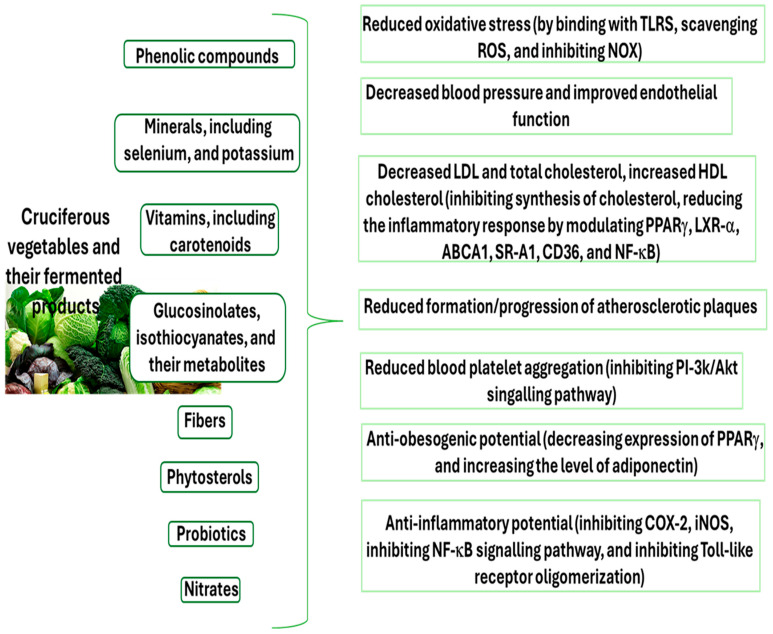
The main components of cruciferous vegetables and their fermented products with cardioprotective potential.

The main components with cardioprotective potential in cruciferous vegetables are summarized in Figure 1, together with their mechanisms, but the demonstrated mechanisms are proposed rather than clinically confirmed. For example, the cardioprotective capacity of these components, especially phenolic compounds, seems to be associated with their antioxidant activity. These components have been found to inhibit ROS production and lipid peroxidation, and to stimulate the activity of various antioxidant enzymes. Moreover, phenolic compounds have anti-platelet properties, especially anti-aggregatory action. They also exhibit significant inhibition against cyclooxygneae-2 (COX-2), inducible nitric oxide synthase (iNOS), interleukin-6 (IL-6), tumor necrosis factor-α (TNF-α), and nuclear factor kappa-light-chain-enhancer of activated B cells (NF-κB) (in vitro and in vivo studies) [86,87,88,89]. Recently, Cabrera-Fuentes et al. [90] have demonstrated that extracellular RNA drives TNF-α-mediated cardiac I/R injury and provides important mechanistic insights into inflammatory pathways that may be modulated by bioactive compounds from cruciferous vegetables. Therefore, TNF-α signaling may be a therapeutic target for cardioprotection.

In addition, Kang et al. [89] noted that wasabia japonica also has anti-inflammatory activity via inhibiting the NF-κB signaling pathways in vitro, but authors did not describe the phytochemical characteristic of the used extract.

Anti-inflammatory action of glucoraphanin by inhibiting Toll-like-receptor oligomerization, and consequent NF-κB activation and Th1/Th17 polymerization was found (in vitro and in vivo models) [61,62,63,64]. Moreover, glucoraphanin had anti-platelet action through the inhibition of the phosphoinositide 3-kinase (PI3k)/protein kinase B (Akt) pathway (ex vivo and in vitro models) [66,67].

For example, Saluk et al. [45] suggest that there could be two ways for red cabbage anthocyanins in the blood platelet to set off the protection mechanism, by their antioxidant properties and directly by binding with Toll-like receptors (TLRs) (in vitro).

## 5. Changes in the Content of Phenolic Compounds and Glucosinolates During Vegetable Processing

### 5.1. Phenolic Compounds

Different processing methods (e.g., boiling, steaming, fermentation, freezing) may change the content of active compounds in cruciferous vegetables. For example, during fermentation, various factors may change the content of phenolic compounds, especially those in complex forms [83]. However, only few data describes changes in the level of phenolic compounds in cruciferous vegetables. In addition, these results vary widely. Cai et al. [91] noted an increase in total content of these compounds in fermented broccoli puree (about 83%) compared to raw broccoli. Similar results were reported by Michalak et al. [92], who compared the content of genistic acid in raw and fermented curly kale leaves. Results of Fang et al. [93] also demonstrate an increase in free phenolic acid content (including gallic, vanillic, caffeic, sinapis, ferulic and other acids) in fermented potherb mustard. In another experiment, Harbaum et al. [94] note more flavonoid derivatives with lower molecular mass, hydroxycinnamic aglycones, and flavonoids in fermented products than in fresh leaves of Pak Choi and Chinese leaf mustard.

On the other hand, a decrease in total phenolic acids was found in various fermented products from cruciferous vegetables. According to Wiczkowski et al. [95], the content of anthocyanins decreased by 24% during the fermentation of red cabbage. Odongo et al. [82] also observed a 75% decrease in phenolic compounds in fermented Ethiopian kale, but the decrease depended on the type of phenolic compounds. For example, more complex compounds were degraded easily.

Szutowska et al. [96] determined the changes in phenolic compounds, vitamin C content and antioxidant properties during spontaneous fermentation of curly kale juice. They observed that total phenolic content and antioxidant properties increased from 48 to 116 mg gallic acid, equivalent to 100 mL, and from 4.5 to 6.8 mM Trolox/100 mL, respectively. However, the content of vitamin C decreased in this fermented product.

Importantly, cooking and other processing (for example, steaming and microwave) of cruciferous vegetables affects their phytochemical composition and bioavailability of various chemical compounds, including phenolic compounds [31,87,88,97,98]. For example, enhanced phenolic compound content was noticed upon boiling in Brussels sprouts [97].

Recently, Uvaraj et al. [99] have compared the bioactive efficacy of cooked and fresh (uncooked) stems and florets of broccoli extracted with three various solvents (aqueous, methanol, and acetonitrile extracts). They studied the antioxidant potential of these extracts using DPPH, 2,2′-azino-bis(3-ethylbenzothiazoline-6-sulfonic acid (ABTS), and metal ion reduction assays. Aqueous and acetonitrile extracts exhibited higher antioxidant properties than methanolic extracts in all used antioxidant assays. The acetonitrile extract also demonstrated the highest anti-inflammatory action (measuring by the albumin denaturation assay). Moreover, increased antioxidant potential was noted in fresh florets and boiled broccoli stems. Authors have also found that the content of total phenolic compounds is higher in the methanolic extract than in the aqueous extract. Similarly, Wang et al. [100] reported that the methanolic extract of broccoli has increased total phenolic compound and antioxidant properties compared to the water extract and acetate extracts. In kale, all processing methods such as microwaving, boiling, stir-frying, and steaming increased the level of total phenolic compounds, but steaming yielded the highest antioxidant potential and content of phenolic compounds. On the other hand, steaming lead to a 35% reduction in the content of these compounds in red cabbage [101].

Recently, Kamchen et al. [102] have analyzed the effect of selenium biofortification on the increase in the content of total phenolic compounds in cruciferous vegetables. However, there is no consensus regarding whether it increases or not.

### 5.2. Glucosinolates

Processing techniques may also change the content of glucosinolates in cruciferous vegetables. For example, significant losses of glucosinolates are caused by grinding, heat treatment or pickling. Losses range from 18.1 to 59.2% [103,104]. For example, cooking in boiling water has a high level of glucosinolate losses, but the losses are lower in steam cooking [105]. Moreover, cooking time affects the concentration of these compounds and their bioavailability. Song and Thornalley [57] noted that boiling cauliflower, cabbage, and broccoli for 30 min led to a progressive decrease in the concentration of glucosinolates. The losses caused by cooking also differ according to the types of cruciferous vegetables (the loss for boiling (for 30 min) cauliflower and broccoli is 75–77%, for boiling (for 30 min) Brussels sprouts is 58%) [57].

It is important that cooking denatures the myrosinase found in cruciferous vegetables, with long cooking and high temperature (˃80 °C) increasing the intensity of denaturation [52,106]. For example, Oliviero et al. [107] observed that consuming cooked fresh broccoli retains small myrosinase activity that can generate higher concentrations of glucoraphanin.

Freezing has also been demonstrated to result in higher retention of glucosinolates compared to refrigeration. For example, storage of broccoli at 6 °C (for 35 days) induced a glucoraphanin loss of 29%, compared to losses of approximately 13% after freezing at −18 °C (for 60 days) [104].

In addition, during fermentation of cruciferous vegetables, various glucosinolates are hydrolyzed and undergo different additional transformations. In these fermented products, glucosinolates are absent or present in small amounts in a native form. During fermentation, the degree of hydrolysis of glucosinolates depends on their microbiological and chemical stability [83,108]. For example, Ciska et al. [108] identified 12 breakdown products of aryl and aliphatic glucosinolates in fermented cabbage. Sinigrin was the most abundant glucosinolate, and it was hydrolyzed to allyl isothiocyanate during fermentation.

The literature body also suggests that during fermentation of cruciferous vegetables, glucosinolates–glucobrassicin is degraded to indole-3-carbinol. About 50% of indole-3-carbinol is converted to diindolylmethane within 1 h after oral administration of indole-3-carbinol. Indole-3-carbinol and diindolylmethane (a major metabolite or condensation product of indole-3-carbinol) have a wide range of health benefits [54,109]. For example, indole-3-carbinol and its major metabolite have various biological activities, including cardioprotective ones. For example, they inhibit blood platelet aggregation and thrombosis in rats [110,111].

Effect of selected processing methods on the content of phenolic compounds and glucosinolates in cruciferous vegetables are shown in Table 3. It is important to note that fermentation especially may have different effects on phenolic compounds content depending on the cruciferous vegetable. It should also be emphasized that the choice of cruciferous vegetables and their fermentation play an important role, as both of these factors affect the amount of phenolic compounds consumed.

## 6. Conclusions

Numerous studies indicate that both extracts from cruciferous vegetables and their active individual components appear to have cardioprotective potential. However, there is no concrete clinical evidence for the efficacy, absorption, and bioavailability of various components with cardioprotective action in cruciferous vegetables. On the other hand, Kempin [112] noted that supplementation with cruciferous vegetable phytochemicals (such as indole-3-carbinol and others (230–450 g/day) is safe. In vivo studies, especially in human models, are needed, as cardioprotective properties in vitro do not equally guarantee this activity after ingestion. Therefore, it is a hot topic in future studies. Moreover, comparing the results of different studies can be hard—even if the concentrations of extracts from cruciferous vegetables and their bioactive compounds, including phenolic compounds and glucosinolates, are the same, the outcomes can be different depending on extraction method, or methods used to assess the cardioprotective activity. Furthermore, cardioprotective properties should be studied with many methods. In addition, standardization of extraction methods and biological assays would make comparing various studies easier.

It is difficult to separate the action of phenolic compounds or glucosinolates from other compounds when determining the results of food-based cruciferous vegetable interventions. Further, when these vegetables are consumed as part of a diet, there may be other factors influencing the effect of active compounds consumed (for example, influences on metabolism and gut microbiota). For example, existing metabolomics have found the importance of dietary vegetables as modulators in various diseases. Recently, He et al. [113] have noted that indole-3-carbinol (the derivative of glucobrassicin; 50–100 mg/kg/day) ameliorates progression of atherosclerosis through remodeling the gut microbiome and metabolomics in high-choline fed ApoE-/- mice (n = 140). Therefore, metabolomics profiling may be a significant tool to better understand the importance of dietary cruciferous vegetables for health of the cardiovascular system.

Further research is also needed to develop effective formulations, including supplements, especially during their long supplementation. With their unique combination of bioactive components and cardioprotective actions, cruciferous vegetables may represent an exciting frontier in plant-based medicine and nutraceutical development.

Because various processing methods, especially fermentation, can affect the chemical content of cruciferous vegetables in different ways, it is important to understand how such methods can affect the content of bioactive compounds (including phenolic compounds and glucosinolates) in these vegetables, to optimize their cardioprotective efficacy in further studies.

## Figures and Tables

**Table 1:** Common names of selected cruciferous vegetables and their parts used for human consumption.

Common Name of Cruciferous Vegetable	Part Used for Human Consumption
Broccoli	inflorescence
Cauliflower	inflorescence
Cabbage	leaves
Kale	leaves
Arugula	leaves
Cress	leaves
Brussels sprouts	buds
Turnip	root
Horseradish	root
Radish	root

**Table 2:** Cardioprotective potential of preparations of cruciferous vegetables and their bioactive compounds in various models.

Preparations of Cruciferous Vegetables and Their Bioactive Compounds	Biological Activity	References
**In vitro models**		
Kaemperol glycosides (0.5–2 mM) isolated from methanolic extract of *E. sativa* leaves	Antioxidant potential—washed human blood platelets (5 × 10^8^/mL)	[42]
Cold-water extract (riches in flavonoids and polyphenolics) from cauliflower	Antioxidant potential	[43]
Caffeic acid and ferulic acid (isolated from kale). Total contents: 4269 and 4887 ng/g fresh weight, respectively	Antioxidant potential measured by DPPH scavenging capacity	[44]
Chloroform fraction from leaves of *R. sativus* (1–400 µg/mL)	Anti-inflammatory potential in LPS-stimulated RAW264.7 cells	[86]
Radish extract (10–100 µg/mL)	Anti-inflammatory potential in LPS-stimulated macrophages	[87]
Red cabbage anthocyanins (0.15–1.5 µg/mL)	Anti-platelet potential—washed human blood platelets	[47]
Red cabbage anthocyanins (0.15–1.5 µg/mL)	Antioxidant potential in human plasma and human blood platelets	[45,46]
Aqueous extract from *E. sativa* leaves (0.1–1 mg/mL)	Anti-platelet potential in human blood platelets	[51]
Glucoraphanin (1–4 µM)	Anti-inflammatory potential in endothelial cells	[61]
Glucoraphanin (1–10 µM)	Antioxidant potential in endothelial cells	[62]
Glucoraphanin (2.5–10 µM)	Anti-inflammatory potential in endothelial cells	[63]
Glucoraphanin (1.25 and 2.5 µM)	Anti-platelet potential in human blood platelets	[66]
Glucoraphanin (15–75 µM)	Anti-platelet potential in mouse blood platelets	[67]
**Animal models**		
Phenolic-rich extract of cabbage (800 mg/kg/day, 8 days)	Antioxidant and antihyperlipidemic potential in Wistar rats (n = 5)	[48]
Methanolic extract from *E. sativa* leaves (1–30 mg/kg/day)	Antihypertensive potential in rats (n = 7)	[49]
Aqueous extract from *E. sativa* leaves (200 mg/kg/day)	Anti-platelet potential in murine model (n = 8)	[51]
Dry powder of red cabbage microgreens (1.09%, for 8 weeks). Anthocyanins and glucosinolates were major bioactive compounds	Hypolipidemic potential in mice fed a high-fat diet (n = 60)	[63]
Tuscan black cabbage sprout extract (enriched in glucosinolates) (15 mg/kg/day, for 21 days)	Hypolipidemic potential in rats fed a high-fat diet (n = 6)	[72]
Glucoraphanin (10 µmol/kg/day, 4 months)	Normalizing blood pressure in hypertensive rats (n = 6)	[75]
Glucoraphanin (0.25 mg/kg/day, for 4 weeks)	Hypolipidemic potential in a rabbit model of hypercholesterolemia	[65]
Glucoraphanin (0.25 mg/kg/day, for 4 weeks)	Antioxidant, anti-inflammatory and hypolipidemic potential in hypercholesterolemic rabbits (n = 5)	[65]
Glucoraphanin (0.125 and 0.250 mg/kg)	Anti-platelet potential in mice (n = 6)	[67]
Glucoraphanin (5 mg/kg/day, for 2 weeks)	Anti-obesity potential in obese mice (n = 8)	[68]
Phenethyl isothiocyanate (30 and 75 mg/kg/day, for 12 weeks)	Anti-inflammatory and hypolipidemic potential in C57BL/6 mice (n = 10)	[77]
**Human models**		
Fresh broccoli sprouts (100 g/day, for one week)	Antioxidant and hypolipidemic potential in healthy human subjects (n = 6)	[19]
Broccoli (21.6 ± 1.6 µmol/dw glucoraphanin and 4.5 µmol/g dw glucoiberin) (400 g per week, for 12 weeks)	Hypolipidemic potential in healthy volunteers (n = 130)	[71]
Four capsules of the activated broccoli seed extract BroccoMax^®^ (equivalent to 32 mg of glucoraphanin, per day, for 2 weeks)	Normalizing blood pressure in women with pregnancy hypertension (n = 12)	[74]
Dried broccoli sprouts (10 g/day, for 4 weeks). The sprouts had a glucoraphanin content of 25.9 ± 8.5 µmol/g dw, and a total glucosinolate content 48.5 ± 14.2 µmol/g dw)	No anti-inflammatory potential in humans with hypertension (n = 40)	[76]
Broccoli extract (enriched in glucosinolates) in tablets (5 and 10 g/day, for 4 weeks)	Antioxidant and hypolipidemic potential in patients with type 2 diabetes (n = 25)	[73]

**Table 3:** Effect of selected processing methods on the content of phenolic compounds and glucosinolates in cruciferous vegetables.

Studied Material	Processing Method	Content of Phenolic Compounds	Content of Glucosinolates	References
Broccoli puree	Fermentation	Increase	No data	[91]
Curly kale leaves	Fermentation	Increase	No data	[92]
Potherb mustard	Fermentation	Increase	No data	[93]
Pak Choi and Chinese leaves mustard	Fermentation	Increase	No data	[94]
Curly kale juice	Fermentation	Increase	No data	[96]
Red cabbage	Fermentation	Decrease	No data	[95]
Ethiopian kale	Fermentation	Decrease	No data	[82]
Brussels sprouts	Boiling	Increase	No data	[97]
Cauliflower, cabbage, and broccoli	Boiling	No data	Decrease	[57]
Red cabbage	Steaming	Decrease	No data	[101]

## Data Availability

No new data were created or analyzed in this study.

## Abbreviations

ABCA1—ATP binding cassette subfamily A member 1; ABTS—2,2′-azino-bis(3-ethylbenzothiazoline-6-sulfonic acid; Akt—protein kinase B; AMPK—AMP-activated protein kinase; CD36—cluster of differentiation 36; CVDs—cardiovascular diseases; DPPH—2,2-diphenyl-1-picrylhydrazyl; FAO—Food and Agriculture Organization; ICAM-1—intracellular adhesion molecule 1; IL-6—interleukin-6; iNOS—inducible nitric oxide synthase; LDL—low-density lipoprotein; LPSs—lipopolysaccharides; LXR-α—liver-X-receptor α; MDA—malondialdehyde; NF-κB—nuclear factor kappa-light-chain-enhancer of activated B cells; NOX—NADPH oxidase; ORAC—oxygen radical absorbance capacity; PI-3k—phosphoinositide 3-kinase; PPARγ—peroxisome proliferator-activated receptor gamma; ROS—reactive oxygen species; SR-A1—scavenger receptor A1; TAC—total antioxidant capacity; TLRs—Toll-like receptors; TNF-α—tumor necrosis factor-α; VCAM-1—vascular cell adhesion protein 1.
